# Heatstroke and Allied Conditions

**Published:** 1919-09-27

**Authors:** 


### HEATSTROKE AND ALLIED CONDITIONS.

### Types Seen in the Eastern Campaigns.

TRUE heatstroke has been most in evidence in Mesopotamia, where one met every form from the severest downwards, also in Macedonia, where the hot summers have claimed a number of victims, but in Egypt and Palestine true heatstroke has not been common, possibly due to the relative dryness of the atmosphere. In East Africa, too, in spite of the extreme frequence of heat exhaustion and sun headaches, instances of really dangerous heatstroke were noticeably rare, and in this area the explanation is probably that, in spite of the humidity of the coastal region, the sun has never the intense power experienced in Mesopotamia and Egypt during the summer months.

### NOTES ON ETIOLOGY.

There is at home some slight confusion concerning the exact meanings of a number of conditions which fall under the category of the "effects of heat," and a brief review of the etiology may be useful in clearing up some of those small points. Heatstroke is probably due both to auto-intoxication, secondary to a lack of escape of heat from the body, following insufficient evaporation from the skin, and to the effects of muscular fatigue. There is markedly deficient oxygenation of the blood and the toxic substances may have a selective action on nerve cells, but a view is also held that the condition is a true tissue acidosis, there being retention of carbonic and lactic acids and a depletion of alkali content in the blood. In any event, the fact remains that relative humidity with its effect on skin evaporation is a most potent factor. The severe form was apparently always associated with sunstroke, and the effect on meninges and eyes. The two may, indeed, be pathologically identical, accounting for much confusion in the nomenclature, but the matter still remains undecided, although opinion inclines towards allowing a sunstroke, apart from heatstroke, while under field conditions the two almost always occur together. In the milder forms, such as heat exhaustion, if the man is relieved of his load and rests recovery is rapid. It is merely a syncopal attack which precedes heat prostration.

### HEATSTROKE PROPER.

Heatstroke proper is worthy of more detailed description. An initial sign is the desire to micturate frequently, followed later by dryness of the skin, drowsiness, giddiness, headache, and marked intolerance of light. The pulse is first rapid, then becoming irregular, the skin feels hot and dry. while the man is markedly pyrexial. The attack is often of rapid onset, and delirium, coma, or convulsions may rapidly ensue. In official records three main types have been differentiated.

(*a*) *Asphyxial.*—The man staggers on with fixed stare, contracted pupils, face cyanosed, until he suddenly collapses senseless and apparently dead. Breathing practically ceases, the pulse is just perceptible.

(*b*) *Paralytic*, with a somewhat similar onset, but ending in deep coma, recurring convulsions, vomiting, diarrhœa, and hyperpyrexia.

(*c*) *Psychopathic*, which is the least fatal, but in which the man's mental balance is upset to an extreme or moderate degree.

### PROPHYLAXIS.

A number of obvious measures are indicated, but under war conditions the majority have not been easy to maintain. It is suggested that sniffing water and vinegar and damping this mixture on the face may help to stimulate the respiratory reflexes. Green or tinted glasses have been successfully employed, but beyond everything simple health measures, combined with protection of neck and occiput from the sun, the carrying of minimum accoutrements, and careful regulation of time and order of marching have been the only measures generally practicable for employment.

### TREATMENT.

Treatment has, of course, varied with the severity of the condition. For true heatstroke, however, the value lay as much in the speed with which assistance was rendered as in the actual measures employed. For the asphyxial type artificial respiration, employed if necessary up to two hours, was the only remedy for which marked success could be claimed; but with the paralytic cases a greater variety of means presented tnemselves. The three main indications were to reduce the temperature, to get rid of the toxins, and to ward off cardiac failure. Cold packing until the rectal temperature registered 102° F., thereafter wrapping the patient in blankets with hot bottles to trunk and limbs and ice to the head was often followed, if perspiration set in, by a gradual recovery. The process was often repeated, and in Mesopotamia, to allow for the frequency of such cases, numerous bath stations were established. Men with a temperature above 103° F. were at once placed in a tub of water at a level sufficient to cover all but the head. Ice was added to the water and friction applied to the limbs until the temperature fell to 102° F., after which the case was continued as above. Cardiac stimulants were given freely, and later chloral enemata, scopolamine, morphia, or bromide were useful in controlling convulsions or wildly delirious cases.

An important factor was the direct attack on tissue acidosis. In those instances where cold treatment did not result in immediate response venesection, followed by slow intravenous 2 per cent. sodium bicarbonate solution, had markedly good results Also the practice of performing lumbar puncture to withdraw about 20 cc. of cerebro-spinal fluid had a remarkable good effect on many cases, and it seems that this is one of the best and most rapid ways to obtain reaction in those which fall under the category of our formerly accepted "sunstroke" cases.

